# Development of a nomogram for identifying prevalent medication discrepancy in maintenance hemodialysis patients: a multicenter cross-sectional study

**DOI:** 10.3389/fmed.2026.1900963

**Published:** 2026-09-28

**Authors:** Xiaodan Lin, Mianzhou Liu, Zhaochuan Li, Yinghua Wu, Hao Guo, Siying Ouyang, Wendi Shao, Shutian Chen, Shihua Zheng, Yancao Li

**Affiliations:** 1 Department of Nursing, Shantou University Medical College, Shantou, China; 2 Department of General Surgery, Shantou Central Hospital, Shantou, China; 3 Department of Hemodialysis, The First Affiliated Hospital of Shantou University Medical College, Shantou, China; 4 Department of Hemodialysis, The Second Affiliated Hospital of Shantou University Medical College, Shantou, China; 5 Department of Hemodialysis, Shantou Chaonan Minsheng Hospital, Shantou, China

**Keywords:** internal-external validation, maintenance hemodialysis, medication beliefs, medication discrepancy, medication reconciliation, nomogram

## Abstract

**Objectives:**

Medication discrepancy reflects an observed mismatch between a prescribed medication regimen and actual medication use and is conceptually distinct from medication nonadherence or medication error. We estimated the prevalence of medication discrepancy in maintenance hemodialysis (MHD) patients and developed a development-stage nomogram for identifying prevalent medication discrepancy.

**Methods:**

This multicenter cross-sectional study included 328 MHD patients from four tertiary-hospital hemodialysis centers in Guangdong Province, China, between December 2022 and May 2023. Medication discrepancy was ascertained with an order-referenced medication-reconciliation process based on the modified Medication Discrepancy Tool. The primary model included three predictor domains with complete data: residential area, monthly income, and the Beliefs about Medicines Questionnaire (BMQ) total score, represented by five model parameters after categorical coding and spline transformation. BMQ was modeled continuously with a three-knot restricted cubic spline. Model performance was evaluated using 1,000-resample bootstrap optimism correction and leave-one-center-out internal-external validation.

**Results:**

Medication discrepancy was identified in 256 of 328 participants (78.05%). BMQ showed an overall nonlinear association with prevalent medication discrepancy (global *P* < 0.001; test for nonlinearity *P* = 0.011). In the primary model, urban residence had an odds ratio (OR) of 1.93 (95% CI 0.99–3.75; *P* = 0.053) versus rural residence, while monthly income >5,000 CNY had an OR of 0.33 (95% CI 0.15–0.73; *P* = 0.006) versus <3,000 CNY. The apparent AUC was 0.723 (95% CI 0.663–0.783) and the bootstrap optimism-corrected AUC was 0.701. Leave-one-center-out validation yielded a pooled AUC of 0.601, Brier score of 0.180, calibration intercept of 0.722, and calibration slope of 0.362, with substantial heterogeneity across centers.

**Conclusion:**

The three-domain nomogram showed moderate apparent discrimination but substantially attenuated and heterogeneous cross-center performance. It should be regarded as a development-stage identification model for prevalent medication discrepancy and requires independent external validation, and potentially recalibration, before clinical use.

## Introduction

Chronic kidney disease requiring maintenance hemodialysis (MHD) is characterized by long-term treatment and frequent multimorbidity, including cardiovascular disease, diabetes, anemia, mineral and electrolyte disorders, and sleep problems (1, 2). Medication-taking is therefore a central component of care (3). In one US hemodialysis cohort, the average pill burden was 19 pills per day, and one-quarter of patients took 25 or more (4). Medication burden is clinically relevant, and potentially inappropriate medication classes have been associated with mortality among older adults initiating hemodialysis (5).

Medication-related harm is a major patient-safety concern, with medication errors estimated to cost approximately US$42 billion globally each year (6, 7). Medication nonadherence, which is related to but conceptually distinct from medication discrepancy, is also common in hemodialysis populations and has shown wide prevalence estimates across studies (8).

Medication-taking behavior in kidney failure is influenced by multiple patient-level factors, and medication adherence remains a recognized challenge (9). Previous nomogram studies have used routinely available clinical information to model medication nonadherence in hemodialysis or chronic kidney disease populations (10, 11).

References 10 and 11 are prior nomogram studies of medication nonadherence in hemodialysis or chronic kidney disease populations. The present study addresses a different outcome: medication discrepancy identified at the same cross-sectional assessment through comparison of the prescribed regimen with actual medication use. This distinction is important because a medication discrepancy should not automatically be interpreted as nonadherence, medication error, or preventable medication-related harm.

Therefore, this study aimed to (1) describe the prevalence of medication discrepancy among patients receiving MHD; (2) examine factors associated with prevalent medication discrepancy; and (3) develop and internally evaluate a nomogram for cross-sectional identification of prevalent medication discrepancy.

## Methods

### Study design and participants

This multicenter cross-sectional study included 328 MHD patients recruited from four hemodialysis centers in three cities in Guangdong Province, China, between December 2022 and May 2023. All participants had chronic kidney disease diagnosed by experienced nephrologists and were receiving regular hemodialysis. Inclusion criteria were: (1) aged ≥18 years; (2) MHD treatment at the participating center for at least 3 months; (3) clear consciousness, adequate cognition, and ability to communicate; and (4) voluntary participation after informed consent. Exclusion criteria were transfer to another center during the survey period, inability to communicate or participate, and current or previous participation in a health-related clinical drug trial. All 328 enrolled participants completed the cross-sectional assessment. All patients were diagnosed with chronic kidney disease by at least two experienced nephrologists and received hemodialysis treatment regularly following the guidelines (12).

The cohort had been completed before the prediction-model analysis, and its sample size had not been prospectively determined specifically for prediction-model development. Earlier subjects-per-variable rules were developed for settings such as linear regression and are not used to justify adequacy for the present binary prediction model (13). We therefore report the fixed available cohort (*N* = 328; 256 participants with and 72 without medication discrepancy), the three predictor domains represented by five model parameters in the primary model, and the internal and internal-external validation results transparently.

### Data collection and medication-discrepancy outcome

Before formal data collection, a preliminary survey was administered to 15 patients to assess comprehensibility and completion time. Six investigators (LMZ, LZC, GH, WD, OYSY, and WYH) completed two rounds of standardized training. Information was collected in face-to-face interviews. Participants capable of completing the questionnaire independently did so; interviewers read or recorded responses when dialysis access or writing difficulty limited completion. Questionnaires were checked immediately after the interview, and laboratory measurements were retrieved from the hospital information systems for the predefined assessment window.

Medication discrepancy was assessed using the modified Chinese Medication Discrepancy Tool (MDT) (14). The active medication-order list served as the reference prescribed-medication list. Because most medications were self-administered and stored at home, actual medication use was ascertained primarily through structured face-to-face interviews. Participants reported the regimen actually being taken, including medication name, dose, timing, frequency, and route or method of administration. These reports were cross-checked against active medication orders and, when available, linked payment and medication-dispensing records in the hospital information system. When details required clarification, a family member familiar with daily medication use could supplement the medication history, and available medication containers could be inspected. Dispensing records were used as corroborative evidence of medication collection but were not considered proof of actual consumption. The actual-use regimen was then compared item by item with the prescribed regimen. Consistent with the modified MDT, discrepancies and their possible causes could be further clarified through communication with the responsible physician or nurse and review of prescription or medication-education information. A participant was classified as having a medication discrepancy when at least one mismatch was identified between the active prescribed regimen and actual medication use in the assessed domains. The modified Chinese MDT previously demonstrated a total content validity of 0.97 and inter-rater reliability of 0.84 (14); these are instrument-validation properties rather than a study-specific kappa estimate. According to the type of medication discrepancy, the patient could select one or more items (14).

The study did not prospectively classify discrepancies as intentional versus unintentional, and duplicate independent adjudication by two blinded assessors was not performed. Consequently, a study-specific inter-rater kappa coefficient is not available. These limitations are explicitly considered in the interpretation of the outcome.

### Sociodemographic and clinical variables

Collected characteristics included sex, age, employment, education, marital status, residential area, living arrangement, medical payment method, monthly self-payment for medical care, monthly income, dialysis vintage, weekly dialysis frequency, comorbidity count, medication types, number of medications, participation in medication training, self-directed medication learning, and willingness to learn medication-related knowledge. Residential area was coded urban versus rural. Monthly income was categorized as <3,000, 3,000–5,000, and >5,000 CNY per month.

Laboratory measurements were obtained from the hospital Laboratory Information System from the most recent centralized hemodialysis-center examination within the assessment window. Variables included white blood cell count (WBC), red blood cell count (RBC), hemoglobin (Hb), platelet count (PLT), serum potassium (K), sodium (Na), calcium (Ca), phosphorus (P), blood urea nitrogen (BUN), albumin, serum ferritin (SF), triglyceride (TG), predialysis creatinine (Cr), C-reactive protein (CRP), uric acid (UA), and total cholesterol (Tch).

### Questionnaires

The BMQ-Specific includes 10 items covering necessity beliefs (five items) and concern beliefs (five items). Each item is scored from 1 (strongly disagree) to 5 (strongly agree). The BMQ total score used in this study was calculated as necessity-belief score minus concern-belief score, yielding a theoretical range from −20 to 20; higher values indicate stronger perceived necessity relative to concerns (15). The Chinese BMQ-Specific has reported good content validity and internal consistency (15). Accordingly, higher total scores in the present analysis indicate stronger perceived necessity relative to medication concerns.

The Pittsburgh Sleep Quality Index (PSQI) assesses seven components of sleep quality and yields a total score from 0 to 21, with higher scores indicating poorer sleep quality (16). PSQI was included in the descriptive assessment but was not part of the primary model. If the score ranges from 5 to 7, it indicates sleep disturbance, while a score of less than 4 indicates good sleep (16).

### Missing data and data audit

The source dataset was audited before analysis. Medication-discrepancy status, residential area, monthly income, and BMQ were complete for all 328 participants. Laboratory tests were not performed uniformly across participants; values representing tests not performed within the assessment window were treated as missing rather than as observed laboratory measurements. Across the 16 laboratory variables, availability ranged from 270/328 for CRP to 324/328 for hemoglobin, and 80 participants lacked at least one laboratory measurement. RBC was available for 321 participants and UA for 286; both were available for 285 participants.

Laboratory missingness was strongly associated with study center (*P* < 0.001), indicating a structured testing pattern rather than simple data-entry loss. Because laboratory variables were not required for the primary model, no laboratory imputation was used in the primary analysis. The primary three-domain model therefore used all 328 participants. The model extended with RBC and UA was evaluated as a complete-case sensitivity analysis in the 285 participants with both measurements. Participants with and without complete RBC/UA measurements are compared in Supplementary Table S2.

### Ethical considerations

The study was approved by the Medical Ethics Committee of Shantou University Medical College (No. SUMC-2021-82-K) and was conducted in accordance with the Declaration of Helsinki. All enrolled participants were adults with sufficient cognitive and communication capacity to provide informed consent personally. Written informed consent was documented by signature or, when a participant was unable to sign because of physical or writing difficulties, by fingerprint after the consent information had been read aloud. Proxy consent from legal guardians or next of kin was not used.

### Model development and statistical analysis

Continuous descriptive variables are reported as mean (standard deviation) when approximately normally distributed and as median [interquartile range] otherwise; categorical variables are reported as counts and percentages. A parsimonious primary specification was defined using three clinically interpretable predictor domains with complete data for all participants: residential area, monthly income, and BMQ total score. These domains represent contextual, socioeconomic, and medication-belief characteristics, respectively, and were retained based on associations observed in the initial analyses together with model parsimony, clinical interpretability, and feasibility of routine assessment. They were treated as predictors of prevalent medication discrepancy rather than causal determinants. After categorical coding and spline transformation, the three domains contributed five model parameters: one for residential area, two indicator parameters for monthly income, and two spline parameters for BMQ. No further significance-based variable deletion was performed after this specification was established. However, the final predictor set and BMQ functional form were determined during the peer-review reanalysis rather than prospectively prespecified. Because the same dataset informed predictor selection, functional-form assessment, and model estimation, residual model-selection optimism cannot be excluded.

BMQ was retained as a continuous predictor. A linear BMQ term was compared with a restricted cubic spline with three knots at the 10th, 50th, and 90th percentiles of the observed distribution (−5, 2, and 10). A likelihood-ratio test supported nonlinearity (*P* = 0.011), so the spline representation was retained. Residential area was coded urban versus rural, and monthly income was represented by two indicator variables using <3,000 CNY as the reference category. Because the model contained a nonlinear BMQ effect, a single OR is not reported for BMQ across its full range; the adjusted association is displayed graphically relative to BMQ=2.

Model discrimination was quantified using the area under the receiver operating characteristic curve (AUC), overall prediction error using the Brier score, and calibration using the calibration intercept and slope. Internal validation was performed using 1,000 bootstrap resamples with a fixed random seed of 20260812. Within each bootstrap sample, model coefficients were refitted and the BMQ spline knots were re-estimated; however, the predictor set and the decision to model BMQ nonlinearly were held fixed, and the complete predictor-selection and functional-form-assessment processes were not repeated. To assess center-level transportability, leave-one-center-out evaluation was performed by fitting the model in three centers and evaluating it in the held-out fourth center, with the process repeated across all four centers (40). Because the predictor set and functional form had been informed by analyses of the full dataset, neither procedure fully incorporated uncertainty arising from the data-informed model-specification process. Center was evaluated as the unit of transportability rather than being incorporated into the nomogram as an individual-level predictor.

RBC and UA were evaluated only in an extended sensitivity model because these variables had incomplete and center-dependent availability. The core and extended models were compared in the same 285 complete cases using apparent AUC and Brier score, together with pooled leave-one-center-out AUC, Brier score, calibration intercept, and calibration slope. Reporting was checked against the TRIPOD + AI statement and the TRIPOD-Cluster checklist (39, 40). Two-sided *P* < 0.05 was used for inferential tests. The complete model specification, including the intercept, predictor coefficients, categorical predictor coding, restricted-cubic-spline specification for BMQ, and the equation for calculating individual predicted probabilities, is provided in Supplementary Box S1.

## Results

### Participant characteristics and prevalence of medication discrepancy

All 328 participants were included in the primary analysis; center enrollment was 81, 100, 70, and 77 participants in centers A-D, respectively. Medication discrepancy was identified in 256 participants (78.05%), while 72 (21.95%) had no medication discrepancy. The cohort included 186 men and 142 women. The median BMQ total score was 2.00 [IQR −1.00, 6.25]. Participant characteristics are summarized in Table 1, and the analysis flow is shown in Figure 1.

**Figure 1:**
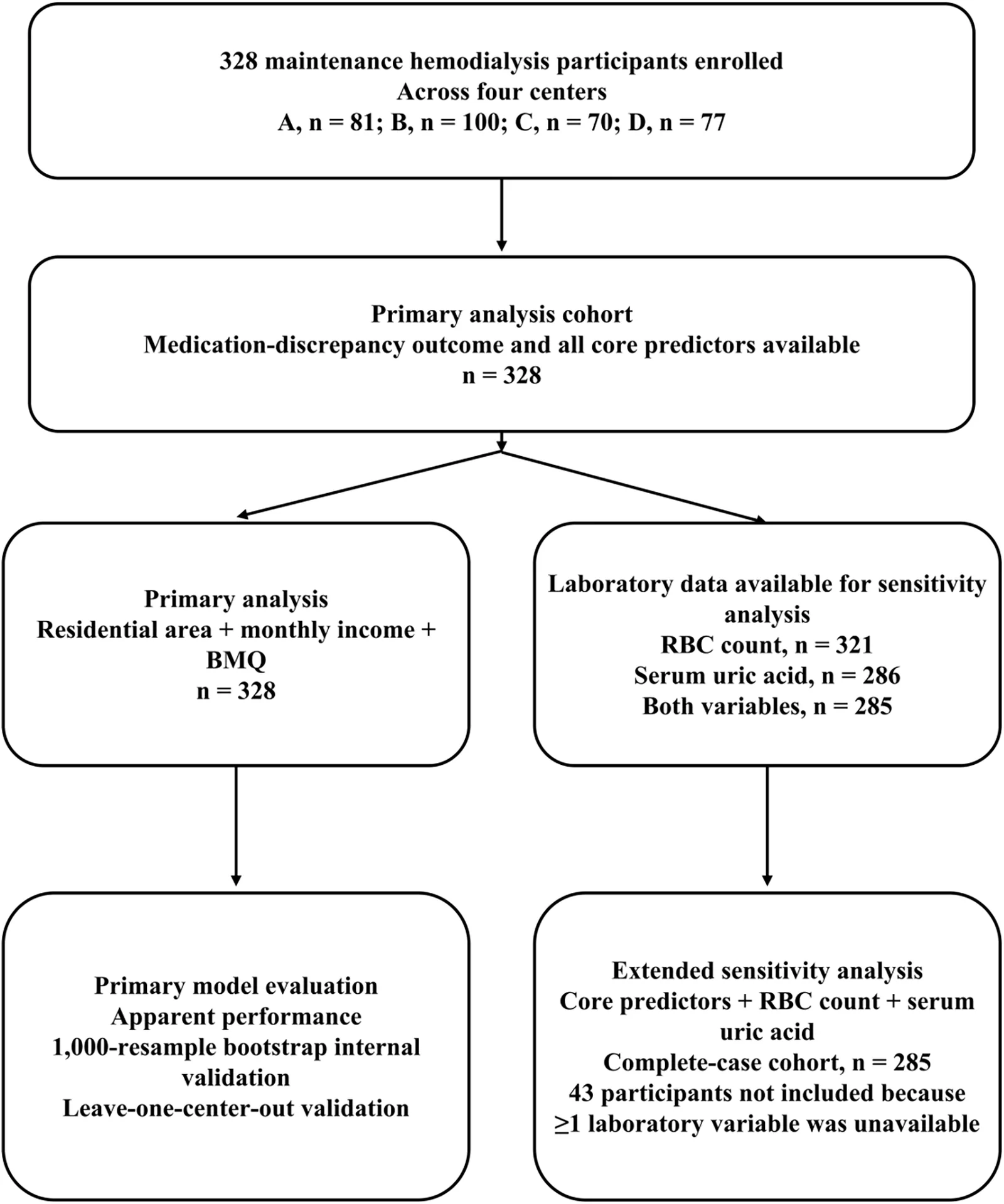
Participant and analysis flow. The primary three-domain model used all 328 participants because the outcome and all core predictors were complete. RBC and uric acid were jointly available for 285 participants and were evaluated only in an extended complete-case sensitivity model.

**Table 1:** Characteristics of participants (*N* = 328).

Characteristic	Total (*N* = 328)	No medication discrepancy (*N* = 72)	Medication discrepancy (*N* = 256)
Gender			
Male	186	42 (58.33)	144 (56.25)
Female	142	30 (41.67)	112 (43.75)
Age			
<45	124	28 (38.89)	96 (37.50)
45–60	137	31 (43.06)	106 (41.41)
≥60	67	13 (18.06)	54 (21.09)
Employment			
Employed	98	24 (33.33)	74 (28.91)
Unemployed	230	48 (66.67)	182 (71.09)
Education level			
Primary school or lower	185	38 (52.78)	147 (57.42)
Junior high school	93	19 (26.39)	74 (28.91)
Senior high school or higher	50	15 (20.83)	35 (13.67)
Marital status			
Married	258	60 (83.33)	198 (77.34)
Single/divorced/widowed	70	12 (16.67)	58 (22.66)
Residential area			
Urban	195	40 (55.56)	155 (60.55)
Rural	133	32 (44.44)	101 (39.45)
Living arrangement			
Alone	17	1 (1.39)	16 (6.25)
With family	311	71 (98.61)	240 (93.75)
Main medical payment method			
National medical insurance	204	39 (54.17)	165 (64.45)
Other payment methods	124	33 (45.83)	91 (35.55)
Self-payment medical cost, CNY/month			
<2,000	152	34 (47.22)	118 (46.09)
2,001–3,000	122	28 (38.89)	94 (36.72)
3,001–5,000	41	7 (9.72)	34 (13.28)
>5,000	13	3 (4.17)	10 (3.91)
Monthly income, CNY			
<3,000	147	27 (37.50)	120 (46.88)
3,000–5,000	101	19 (26.39)	82 (32.03)
>5,000	80	26 (36.11)	54 (21.09)
Dialysis vintage, years			
<3	142	29 (40.28)	113 (44.14)
3–7	107	21 (29.17)	86 (33.59)
>7	79	22 (30.56)	57 (22.27)
Weekly dialysis frequency			
≤2	47	13 (18.06)	34 (13.28)
>2	281	59 (81.94)	222 (86.72)
Comorbidity count			
1–2	106	30 (41.67)	76 (29.69)
3–4	156	31 (43.06)	125 (48.83)
≥5	66	11 (15.28)	55 (21.48)
Medication types			
≤3	56	17 (23.61)	39 (15.23)
4–6	153	29 (40.28)	124 (48.44)
≥7	119	26 (36.11)	93 (36.33)
Number of medications			
≤10	156	34 (47.22)	122 (47.66)
11–20	127	24 (33.33)	103 (40.23)
≥20	45	14 (19.44)	31 (12.11)
Medication training			
Yes	128	29 (40.28)	99 (38.67)
No	200	43 (59.72)	157 (61.33)
Self-taught medication knowledge			
Yes	145	37 (51.39)	108 (42.19)
No	183	35 (48.61)	148 (57.81)
Willingness to learn medication knowledge			
Yes	295	67 (93.06)	228 (89.06)
No	33	5 (6.94)	28 (10.94)
BMQ total score, median [IQR]	2.000 [−1.000, 6.250]	5.000 [3.000, 8.000]	2.000 [−2.000, 6.000]
Necessity beliefs, mean (SD)	18.000 (4.551)	19.708 (4.109)	17.520 (4.561)
Concern beliefs, mean (SD)	15.476 (4.522)	14.417 (4.958)	15.773 (4.355)
PSQI total score, median [IQR]	10.000 [7.000, 13.000]	8.500 [5.000, 12.000]	10.000 [7.000, 13.000]
Laboratory measurements			
WBC, median [IQR]	6.210 [5.203, 7.635]	6.040 [5.150, 7.482]	6.295 [5.210, 7.700]
RBC, mean (SD)	3.841 (0.719)	3.680 (0.735)	3.888 (0.709)
Hb, mean (SD)	107.217 (18.708)	105.333 (19.700)	107.755 (18.420)
PLT, median [IQR]	196.000 [157.000, 239.000]	188.500 [160.000, 236.250]	196.000 [156.500, 239.000]
K, mean (SD)	4.648 (0.934)	4.590 (0.791)	4.665 (0.972)
Na, mean (SD)	137.304 (3.045)	137.566 (3.073)	137.228 (3.040)
Ca, median [IQR]	2.190 [2.020, 2.320]	2.200 [2.085, 2.325]	2.185 [2.020, 2.320]
P, median [IQR]	2.020 [1.682, 2.560]	1.990 [1.623, 2.547]	2.030 [1.690, 2.560]
BUN, median [IQR]	25.100 [16.012, 31.400]	25.515 [15.138, 31.665]	24.890 [16.543, 31.300]
Albumin, mean (SD)	38.648 (4.322)	38.933 (5.199)	38.566 (4.044)
SF, median [IQR]	154.100 [42.700, 356.200]	158.800 [44.930, 381.500]	147.300 [43.025, 354.275]
TG, median [IQR]	1.420 [1.030, 2.305]	1.350 [0.998, 2.167]	1.430 [1.045, 2.310]
Cr, median [IQR]	948.200 [660.650, 1189.250]	931.300 [660.650, 1189.400]	952.300 [667.400, 1188.525]
CRP, median [IQR]	3.340 [1.458, 8.207]	3.340 [1.632, 7.077]	3.340 [1.365, 8.343]
UA, median [IQR]	438.500 [343.500, 508.600]	444.000 [390.450, 524.000]	436.000 [334.000, 507.000]
Tch, median [IQR]	3.730 [3.155, 4.585]	3.670 [3.030, 4.460]	3.730 [3.167, 4.643]

Laboratory summaries are based on available observations; variable-specific denominators and center-specific availability are reported in Supplementary Table S1.

BMQ, Beliefs about Medicines Questionnaire; PSQI, Pittsburgh Sleep Quality Index; IQR, interquartile range; SD, standard deviation.

### Missing-data audit

Variable-specific laboratory availability ranged from 82.3% for CRP (270/328) to 98.8% for hemoglobin (324/328). Eighty participants had at least one unmeasured laboratory test. RBC/UA complete-case status was strongly associated with study center. Medication-discrepancy prevalence did not materially differ between participants with complete RBC/UA data and those without complete data (222/285 [77.9%] vs. 34/43 [79.1%], *P* = 0.862). Characteristics of these groups are shown in Supplementary Table S2, and center-specific laboratory availability is shown in Supplementary Figure S1.

### Primary model

The primary model contained residential area, monthly income, and BMQ total score (Table 2). The graphical score estimates the probability of a medication discrepancy present at the cross-sectional assessment and does not represent prospective risk (Figure 3). BMQ was modeled with a restricted cubic spline using knots at −5, 2, and 10. The overall BMQ association was statistically significant (global *P* < 0.001), and the nonlinear component was supported by the likelihood-ratio test (*P* = 0.011). Compared with rural residence, urban residence had an OR of 1.93 (95% CI 0.99–3.75; *P* = 0.053). Compared with monthly income <3,000 CNY, income 3,000–5,000 CNY had an OR of 0.80 (95% CI 0.39–1.65; *P* = 0.548), and income >5,000 CNY had an OR of 0.33 (95% CI 0.15–0.73; *P* = 0.006). Because BMQ was nonlinear, the spline coefficients are reported without a single overall OR and the adjusted BMQ association is displayed in Figure 2.

**Table 2:** Primary logistic model for prevalent medication discrepancy.

Variable	Level/term	β	SE	*P* value	OR (95% CI)
Intercept	–	1.524	0.277	<0.001	–
Residential area	Rural	Reference			Reference
	Urban	0.657	0.339	0.053	1.93 (0.99–3.75)
Monthly income	<3,000 CNY	Reference			Reference
	3,000–5,000 CNY	−0.221	0.368	0.548	0.80 (0.39–1.65)
	>5,000 CNY	−1.098	0.397	0.006	0.33 (0.15–0.73)
BMQ (restricted cubic spline)	Linear spline basis	−0.355	0.111	0.001	–
	Nonlinear spline basis	0.248	0.108	0.022	–

BMQ was modeled continuously with a restricted cubic spline with knots at the 10th, 50th, and 90th percentiles (−5, 2, and 10). Global BMQ association *P* < 0.001; likelihood-ratio test for nonlinearity *P* = 0.011. A single OR is not reported for the spline because the effect varies across the BMQ range. The model intercept is shown to support full model specification; the complete implementation equation is provided in Supplementary Box S1.

**Figure 2:**
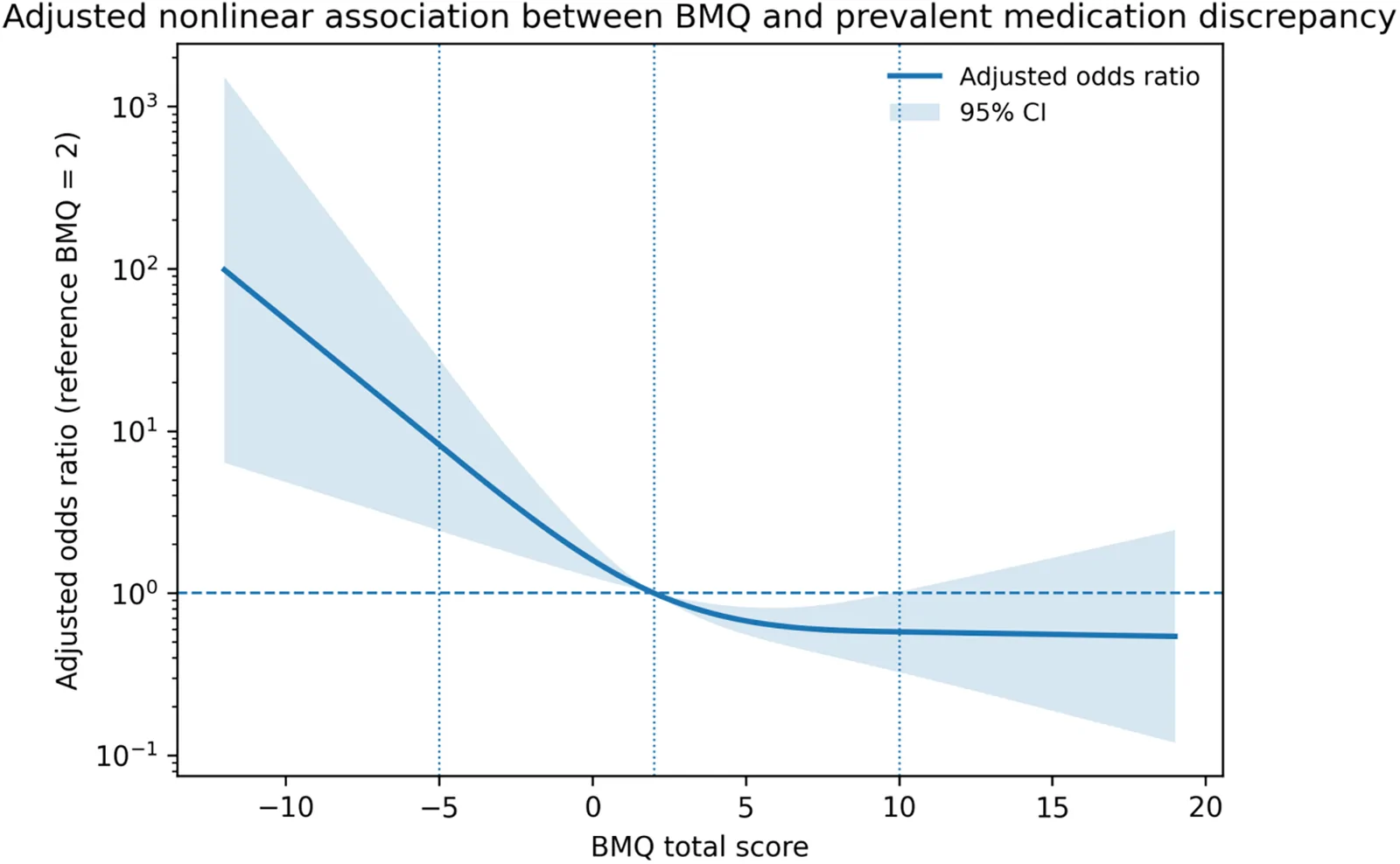
Adjusted nonlinear association between BMQ total score and prevalent medication discrepancy. Odds ratios are shown relative to BMQ=2, with 95% confidence intervals. Vertical dotted lines indicate the spline knots at −5, 2, and 10.

**Figure 3:**
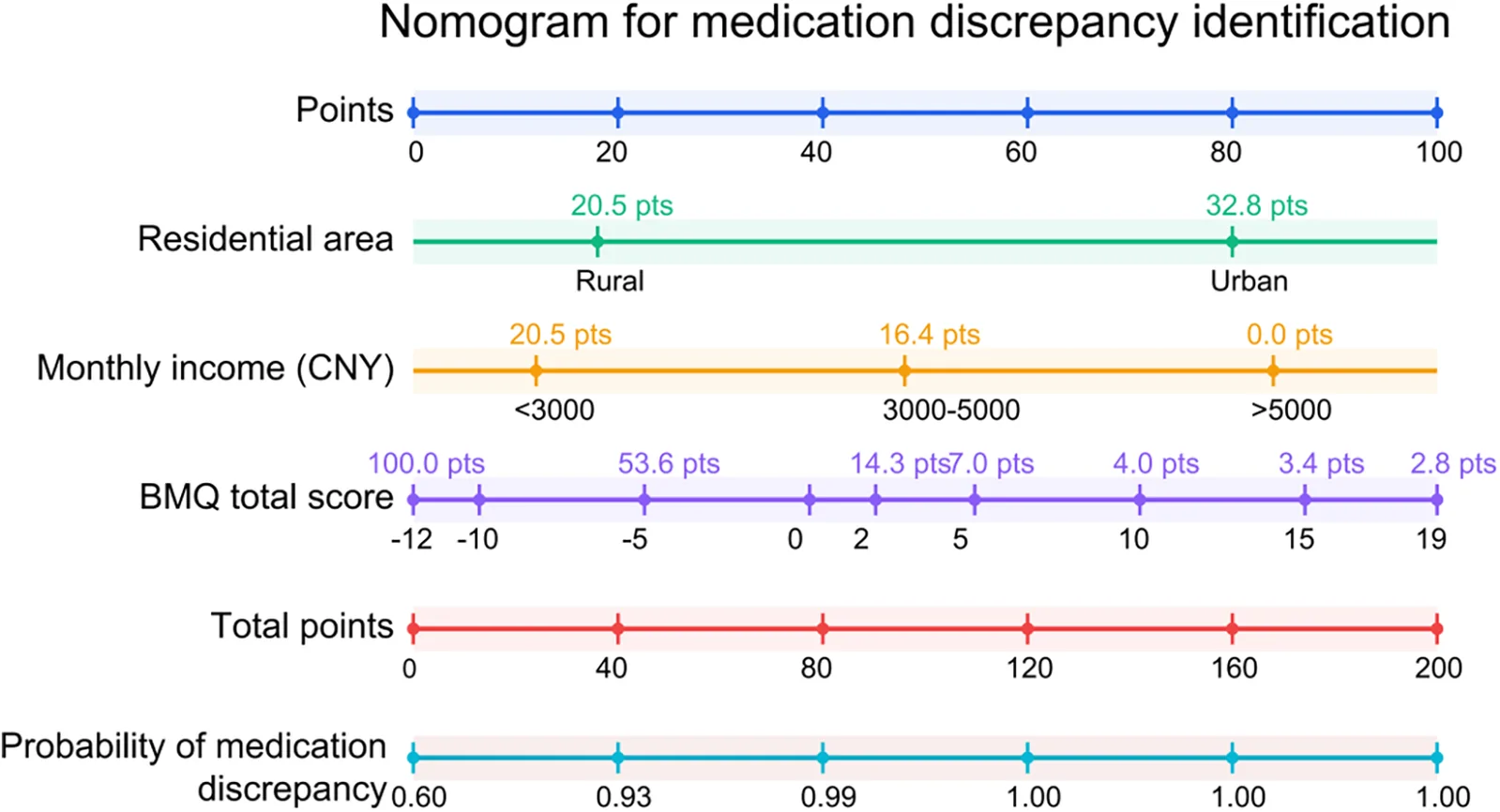
Nomogram representation of the three-domain development model. The BMQ contribution is nonlinear because BMQ was modeled using a restricted cubic spline. The graphical score estimates the probability of a medication discrepancy present at the cross-sectional assessment and does not represent prospective risk.

### Model performance and internal-external validation

The apparent AUC of the primary model was 0.723 (95% CI 0.663–0.783) and the apparent Brier score was 0.154. After 1,000-resample bootstrap optimism correction, the AUC was 0.701, Brier score 0.161, calibration intercept 0.124, and calibration slope 0.867. Leave-one-center-out performance was substantially weaker: the pooled AUC was 0.601, Brier score 0.180, calibration intercept 0.722, and calibration slope 0.362. Center-specific AUCs ranged from 0.462 to 0.789, demonstrating marked heterogeneity across centers (Table 3 and Figure 4).

**Table 3:** Performance of the primary model.

Evaluation set	*N*	AUC	Brier score	Calibration intercept	Calibration slope
Development sample, apparent	328	0.723 (95% CI 0.663–0.783)	0.154	0.000	1.000
Bootstrap optimism-corrected	328	0.701	0.161	0.124	0.867
LOCO pooled predictions	328	0.601	0.180	0.722	0.362
Held-out center A	81	0.628	0.170	1.441	0.230
Held-out center B	100	0.789	0.149	−0.753	1.279
Held-out center C	70	0.462	0.194	1.382	0.059
Held-out center D	77	0.773	0.216	−2.869	1.992

Apparent calibration intercept and slope are 0 and 1 by construction when assessed in the same development data used to fit the model.

LOCO, leave-one-center-out internal-external validation.

**Figure 4:**
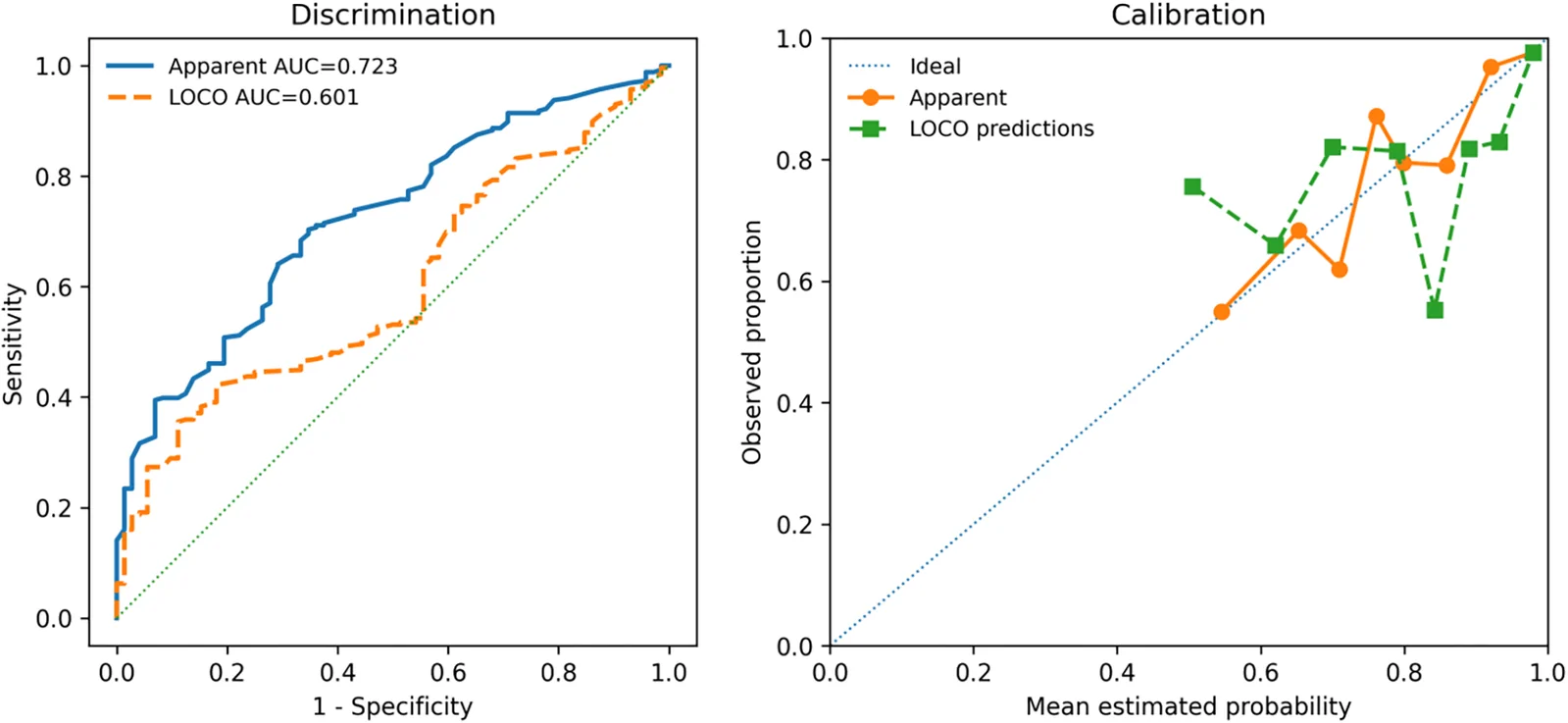
Discrimination and calibration of the primary model. The left panel compares the apparent receiver operating characteristic curve with pooled leave-one-center-out predictions. The right panel shows grouped apparent and leave-one-center-out calibration. Bootstrap optimism-corrected metrics are reported in Table 3.

### Sensitivity analysis with RBC and uric acid

RBC and UA were evaluated only in the 285 participants with both measurements. In this common sample, adding RBC and UA increased the apparent AUC from 0.721 to 0.749 and reduced the apparent Brier score from 0.155 to 0.148. Under leave-one-center-out validation, the pooled AUC increased from 0.576 to 0.601 and the Brier score changed from 0.188 to 0.186. The pooled calibration intercept changed from 0.878 to 0.777, while the calibration slope increased from 0.248 to 0.302. Although these findings indicated a modest numerical improvement, both calibration slopes remained substantially below the ideal value of 1, and changes in center-specific AUC and Brier score were inconsistent. Considering the limited cross-center improvement, center-dependent laboratory availability, and model parsimony, the three-domain model was retained as the primary development model. Detailed comparisons are provided in Supplementary Tables S3 and S4.

## Discussion

In this multicenter cross-sectional cohort, medication discrepancy was identified in 78.05% of MHD patients. The three-domain model combined residential area, monthly income, and a nonlinear continuous BMQ effect. Although apparent discrimination was moderate, bootstrap correction reduced the AUC from 0.723 to 0.701 and leave-one-center-out evaluation reduced it further to 0.601, with substantial center-to-center heterogeneity. The model therefore remains at the development stage and requires independent external validation before clinical use. In the common complete-case sample, adding RBC and UA produced only modest improvements in pooled leave-one-center-out discrimination, overall prediction error, and calibration. Both calibration slopes remained substantially below 1, and center-specific changes were inconsistent, supporting retention of the more parsimonious three-domain model. The selected domains represent contextual, socioeconomic, and medication-belief characteristics that are readily available in routine assessment; their inclusion was intended to balance empirical association, parsimony, interpretability, and feasibility, and the observed associations should not be interpreted as causal.

Medication safety remains an international patient-safety priority (17), while high pill burden contributes to the broader treatment burden experienced by patients with advanced kidney disease (18). A nomogram is a graphical representation of a regression-based model, but its usefulness depends on performance beyond the apparent development sample (19). In the primary model, the apparent AUC of 0.723 decreased to 0.701 after bootstrap optimism correction and to 0.601 under leave-one-center-out validation, arguing against claims of established clinical performance. Medication beliefs remain clinically relevant: greater perceived necessity relative to concerns has been associated with better medication adherence (20), and temporal self-regulation constructs also contribute to adherence behavior (21). In hemodialysis patients, adherence is further shaped by capability, opportunity, and motivation (22). Because the model has not undergone independent external validation, its clinical utility and net benefit remain uncertain and should be assessed in future validation studies using prespecified, clinically relevant threshold probabilities.

Medication reconciliation can identify discrepancies in clinical care (23), and pharmacist-led interventions in hemodialysis have shown potential to improve medication adherence and clinical outcomes (24); patient-centered pharmacist-care strategies are also being evaluated prospectively (25). Medication literacy is another relevant patient-level factor in hemodialysis populations (26). Evidence on nonadherence from other chronic-disease settings suggests socioeconomic and regional differences (27), while studies of urban low-income populations illustrate the influence of access and social context on medication-taking behavior (28). Broader health-system capacity and access also vary across countries and vulnerable urban populations (29, 30). CKD policy, financing, and oversight differ internationally (31, 32), providing important context for the cross-center heterogeneity observed in our study.

Medication complexity and polypharmacy are well-recognized concerns in older and CKD populations (33, 34). Longitudinal research has also described trajectories of medication adherence in chronic disease (35), reinforcing that a cross-sectional discrepancy assessment should not be interpreted as a stable adherence phenotype. Other burdens of CKD, including sleep problems, are managed alongside medication-related demands (36), and the broader CKD literature emphasizes substantial morbidity and the need for integrated care (37). Adherence itself can vary around clinical encounters, as illustrated by the ‘white coat adherence’ phenomenon reported in diabetes (38). These related findings provide context but do not establish that the present cross-sectional nomogram predicts future behavior, treatment response, or clinical outcomes.

### Strengths and limitations

Strengths include the multicenter cohort, audit of source-data denominators and laboratory missingness, explicit modeling of BMQ as a continuous nonlinear predictor, bootstrap optimism correction, and center-level internal-external validation. Several limitations are important. First, the cross-sectional design supports identification of prevalent medication discrepancy only and cannot establish temporal prediction or causality. Second, the final predictor set and functional form were established during the peer-review reanalysis rather than prospectively prespecified. Because the same dataset informed both model specification and estimation, residual model-selection optimism may remain. Although bootstrap resampling and leave-one-center-out evaluation were performed, these procedures did not repeat the complete predictor-selection and functional-form-assessment processes. Consequently, the reported performance estimates may still be optimistic and require confirmation in an independent external cohort. Third, no independent temporal or external validation cohort was available, and cross-center performance was heterogeneous and substantially weaker than apparent performance. Fourth, actual medication use relied primarily on participant report. Although reports were cross-checked with active medication orders and available payment or dispensing records, medication containers, family informants, and clinician clarification, these sources cannot confirm actual consumption, and recall, reporting, and outcome-misclassification bias remain possible. The study also did not prospectively distinguish intentional from unintentional discrepancies or use duplicate independent blinded adjudication; a study-specific kappa coefficient is therefore unavailable. Fifth, laboratory testing was incomplete and center-dependent, so the RBC/UA extension used complete cases without imputation. Finally, the original cohort size was not prospectively calculated for prediction-model development, emphasizing the need for *a priori* sample-size planning in future validation or redevelopment studies.

## Conclusion

Medication discrepancy was common in this multicenter sample of MHD patients. A three-domain nomogram incorporating residential area, monthly income, and nonlinear BMQ showed moderate apparent discrimination but limited and heterogeneous cross-center transportability. The model should remain at the development stage and should not be used as a clinically ready prediction tool. Independent external validation, with recalibration or redevelopment if required, is necessary before evaluating any model-guided medication-reconciliation strategy in practice.

## Data Availability

The datasets generated and/or analyzed during the current study are not publicly available because they contain potentially identifiable patient information and are subject to institutional and ethical restrictions. De-identified data may be made available from the corresponding author upon reasonable request and subject to approval by the relevant ethics committee or institution.
