# Supplementary material for: Development of a nomogram for identifying prevalent medication discrepancy in maintenance hemodialysis patients: a multicenter cross-sectional study
Source: Front Med (Lausanne). 2026 Sep 28;13:1900963. doi: 10.3389/fmed.2026.1900963 (PMC13654690; doi:10.3389/fmed.2026.1900963)
Supplement: Supplementary file 1 [file Datasheet1.docx]

**Supplementary Material**

**Supplementary Table S1. Variable-specific laboratory availability**

| **Laboratory variable** | **Overall n/328** | **Center A n/81** | **Center B n/100** | **Center C n/70** | **Center D n/77** |
| --- | --- | --- | --- | --- | --- |
| WBC | 322 | 81 | 94 | 70 | 77 |
| RBC | 321 | 81 | 93 | 70 | 77 |
| Hb | 324 | 81 | 96 | 70 | 77 |
| PLT | 323 | 81 | 95 | 70 | 77 |
| K | 319 | 81 | 92 | 69 | 77 |
| Na | 301 | 80 | 74 | 70 | 77 |
| Ca | 322 | 81 | 94 | 70 | 77 |
| P | 316 | 80 | 90 | 70 | 76 |
| BUN | 316 | 81 | 93 | 65 | 77 |
| Albumin | 286 | 80 | 62 | 68 | 76 |
| SF | 309 | 80 | 89 | 64 | 76 |
| TG | 311 | 80 | 86 | 69 | 76 |
| Cr | 295 | 81 | 67 | 70 | 77 |
| CRP | 270 | 79 | 46 | 68 | 77 |
| UA | 286 | 80 | 60 | 70 | 76 |
| Tch | 287 | 80 | 62 | 69 | 76 |

Note: A laboratory value was considered missing when the test had not been performed within the predefined assessment window. Eighty of 328 participants lacked at least one of these 16 laboratory measurements.

**Supplementary Table S2. Comparison of participants with and without complete RBC/uric-acid measurements**

| **Characteristic** | **Complete RBC+UA (N=285)** | **Incomplete RBC and/or UA (N=43)** | ***P* value** |
| --- | --- | --- | --- |
| **Medication discrepancy, n (%)** |  |  | 0.862 |
| Yes | 222 (77.9%) | 34 (79.1%) |  |
| **Study center, n (%)** |  |  | <0.001 |
| A | 80 (28.1%) | 1 (2.3%) |  |
| B | 59 (20.7%) | 41 (95.3%) |  |
| C | 70 (24.6%) | 0 (0.0%) |  |
| D | 76 (26.7%) | 1 (2.3%) |  |
| **Gender, n (%)** |  |  | 0.648 |
| Male | 163 (57.2%) | 23 (53.5%) |  |
| Female | 122 (42.8%) | 20 (46.5%) |  |
| **Age, n (%)** |  |  | 0.571 |
| <45 | 109 (38.2%) | 15 (34.9%) |  |
| 45-60 | 116 (40.7%) | 21 (48.8%) |  |
| ≥60 | 60 (21.1%) | 7 (16.3%) |  |
| **Employment, n (%)** |  |  | 0.138 |
| Employed | 81 (28.4%) | 17 (39.5%) |  |
| Unemployed | 204 (71.6%) | 26 (60.5%) |  |
| **Education level, n (%)** |  |  | 0.003 |
| Primary school or lower | 170 (59.6%) | 15 (34.9%) |  |
| Junior high school | 72 (25.3%) | 21 (48.8%) |  |
| Senior high school or higher | 43 (15.1%) | 7 (16.3%) |  |
| **Marital status, n (%)** |  |  | 0.385 |
| Married | 222 (77.9%) | 36 (83.7%) |  |
| Single/divorced/widowed | 63 (22.1%) | 7 (16.3%) |  |
| **Residential area, n (%)** |  |  | <0.001 |
| Urban | 153 (53.7%) | 42 (97.7%) |  |
| Rural | 132 (46.3%) | 1 (2.3%) |  |
| **Living arrangement, n (%)** |  |  | 0.191 |
| Alone | 13 (4.6%) | 4 (9.3%) |  |
| With family | 272 (95.4%) | 39 (90.7%) |  |
| **Main medical payment method, n (%)** |  |  | <0.001 |
| National medical insurance | 199 (69.8%) | 5 (11.6%) |  |
| Other payment methods | 86 (30.2%) | 38 (88.4%) |  |
| **Self-payment medical cost (CNY/month), n (%)** |  |  | 0.443 |
| <2000 | 135 (47.4%) | 17 (39.5%) |  |
| 2001-3000 | 103 (36.1%) | 19 (44.2%) |  |
| 3001-5000 | 37 (13.0%) | 4 (9.3%) |  |
| >5000 | 10 (3.5%) | 3 (7.0%) |  |
| **Monthly income (CNY), n (%)** |  |  | <0.001 |
| <3000 | 137 (48.1%) | 10 (23.3%) |  |
| 3000-5000 | 88 (30.9%) | 13 (30.2%) |  |
| >5000 | 60 (21.1%) | 20 (46.5%) |  |
| **Dialysis vintage (years), n (%)** |  |  | 0.036 |
| <3 | 126 (44.2%) | 16 (37.2%) |  |
| 3-7 | 97 (34.0%) | 10 (23.3%) |  |
| >7 | 62 (21.8%) | 17 (39.5%) |  |
| **Weekly dialysis frequency, n (%)** |  |  | 0.001 |
| ≤2 | 34 (11.9%) | 13 (30.2%) |  |
| >2 | 251 (88.1%) | 30 (69.8%) |  |
| **Comorbidity count, n (%)** |  |  | 0.015 |
| 1-2 | 100 (35.1%) | 6 (14.0%) |  |
| 3-4 | 128 (44.9%) | 28 (65.1%) |  |
| ≥5 | 57 (20.0%) | 9 (20.9%) |  |
| **Medication types, n (%)** |  |  | 0.001 |
| ≤3 | 45 (15.8%) | 11 (25.6%) |  |
| 4-6 | 126 (44.2%) | 27 (62.8%) |  |
| ≥7 | 114 (40.0%) | 5 (11.6%) |  |
| **Number of medications, n (%)** |  |  | <0.001 |
| ≤10 | 119 (41.8%) | 37 (86.0%) |  |
| 11-20 | 122 (42.8%) | 5 (11.6%) |  |
| ≥20 | 44 (15.4%) | 1 (2.3%) |  |
| **Medication training, n (%)** |  |  | <0.001 |
| Yes | 127 (44.6%) | 1 (2.3%) |  |
| No | 158 (55.4%) | 42 (97.7%) |  |
| **Self-taught medication knowledge, n (%)** |  |  | <0.001 |
| Yes | 138 (48.4%) | 7 (16.3%) |  |
| No | 147 (51.6%) | 36 (83.7%) |  |
| **Willingness to learn medication knowledge, n (%)** |  |  | 0.471 |
| Yes | 255 (89.5%) | 40 (93.0%) |  |
| No | 30 (10.5%) | 3 (7.0%) |  |
| BMQ total score | 3.00 [0.00, 7.00] | 0.00 [-6.00, 4.50] | <0.001 |
| PSQI total score | 10.00 [7.00, 13.00] | 8.00 [5.00, 12.00] | 0.008 |

Note: Categorical variables were compared using chi-square tests; BMQ and PSQI total scores are presented as median [IQR] and compared using the Mann-Whitney U test. The comparison is descriptive and is intended to characterize selective laboratory availability rather than to identify causal determinants of missingness. Category labels and grouped definitions in Supplementary Table S2 were re-audited against the corrected source dataset and aligned with manuscript Table 1.

**Supplementary Table S3. Core versus RBC/uric-acid extended model in the common complete-case sample (N=285)**

| **Performance measure** | **Core model** | **Core + RBC + UA** |
| --- | --- | --- |
| Apparent AUC | 0.721 | 0.749 |
| Apparent Brier score | 0.155 | 0.148 |
| Pooled LOCO AUC | 0.576 | 0.601 |
| Pooled LOCO Brier score | 0.188 | 0.186 |
| Pooled LOCO calibration intercept | 0.878 | 0.777 |
| Pooled LOCO calibration slope | 0.248 | 0.302 |

Note: Both models were evaluated in exactly the same 285 participants. Calibration intercept and slope were estimated by logistic recalibration of the observed outcome on the logit of pooled held-out probabilities; ideal values are 0 and 1, respectively. In each LOCO iteration, BMQ spline knots were re-estimated in the three-center training sample and applied to the held-out center. LOCO, leave-one-center-out internal-external validation.

**Supplementary Table S4. Center-specific LOCO performance of core and extended models in complete cases**

| **Held-out center** | **N** | **Core AUC** | **Core Brier** | **Extended AUC** | **Extended Brier** |
| --- | --- | --- | --- | --- | --- |
| A | 80 | 0.634 | 0.169 | 0.569 | 0.163 |
| B | 59 | 0.790 | 0.160 | 0.824 | 0.150 |
| C | 70 | 0.476 | 0.194 | 0.538 | 0.198 |
| D | 76 | 0.725 | 0.225 | 0.684 | 0.226 |

Note: The extended model produced inconsistent center-specific changes in performance, which, together with center-dependent laboratory availability, supported retention of the three-domain core model as the primary development model.

Supplementary Figure S1. Center-specific availability of laboratory variables. Availability reflects whether the measurement was performed within the assessment window.

**Supplementary Box S1. Full model specification and implementation**

The primary model is a logistic model for the probability of prevalent medication discrepancy (outcome coded 1 for discrepancy present and 0 for no discrepancy). Rural residence and monthly income <3,000 CNY are the reference categories. Urban residence is coded U=1 for urban and U=0 for rural. Two income indicator variables are used: I1=1 for monthly income 3,000-5,000 CNY (otherwise 0) and I2=1 for monthly income >5,000 CNY (otherwise 0). BMQ total score is denoted by x and is modeled with a restricted cubic spline using knots at -5, 2, and 10.

Restricted-cubic-spline basis: h1(x)=x. The nonlinear basis is h2(x) = [max(x+5,0)^3 - 1.875×max(x-2,0)^3 + 0.875×max(x-10,0)^3]/225.

Full linear predictor: η = 1.524404 + 0.657458U - 0.220681I1 - 1.098338I2 - 0.354700h1(x) + 0.248293h2(x).

Individual predicted probability: P(prevalent medication discrepancy) = 1/[1 + exp(-η)].

The fitted coefficients before rounding were: intercept 1.524403669; urban residence 0.657457690; monthly income 3,000-5,000 CNY -0.220680940; monthly income >5,000 CNY -1.098337552; BMQ linear spline term -0.354700027; and BMQ nonlinear spline term 0.248292938. This specification can be implemented directly from the equation above and does not require proprietary software.

For bootstrap internal validation, 1,000 resamples were generated using random seed 20260812. Within each resample, model coefficients were refitted and BMQ spline knots were re-estimated; the predictor set and the decision to model BMQ nonlinearly were held fixed. Leave-one-center-out internal-external validation trained the model on three centers and evaluated predictions in the fourth center, repeated for all four centers. The RBC/UA sensitivity analysis was restricted to the common complete-case sample (N=285).
